# Familial Influences on Neuroticism and Education in the UK Biobank

**DOI:** 10.1007/s10519-019-09984-5

**Published:** 2019-12-04

**Authors:** R. Cheesman, J. Coleman, C. Rayner, K. L. Purves, G. Morneau-Vaillancourt, K. Glanville, S. W. Choi, G. Breen, T. C. Eley

**Affiliations:** 1 Social Genetic & Developmental Psychiatry Centre, Institute of Psychiatry, Psychology & Neuroscience, King’s College London, 16 de Crespigny Park, Denmark Hill, London, SE5 8AF UK; 2 NIHR Biomedical Research Centre for Mental Health, South London and Maudsley NHS Trust, London, UK; 3 Research Unit on Child Psychosocial Maladjustment, Laval University, Quebec City, Canada

**Keywords:** Genomics, Family data, Heritability, Neuroticism, Education

## Abstract

Genome-wide studies often exclude family members, even though they are a valuable source of information. We identified parent–offspring pairs, siblings and couples in the UK Biobank and implemented a family-based DNA-derived heritability method to capture additional genetic effects and multiple sources of environmental influence on neuroticism and years of education. Compared to estimates from unrelated individuals, total heritability increased from 10 to 27% and from 17 to 56% for neuroticism and education respectively by including family-based genetic effects. We detected no family environmental influences on neuroticism. The couple similarity variance component explained 35% of the variation in years of education, probably reflecting assortative mating. Overall, our genetic and environmental estimates closely replicate previous findings from an independent sample. However, more research is required to dissect contributions to the additional heritability by rare and structural genetic effects, assortative mating, and residual environmental confounding. The latter is especially relevant for years of education, a highly socially contingent variable, for which our heritability estimate is at the upper end of twin estimates in the literature. Family-based genetic effects could be harnessed to improve polygenic prediction.

**Electronic supplementary material:**

The online version of this article (10.1007/s10519-019-09984-5) contains supplementary material, which is available to authorized users.

## Introduction

Heritability measures the proportion of individual differences in a trait explained by inherited genetic variation, and is traditionally estimated by comparing the resemblance of identical and non-identical twins (Knopik et al. 2017). Researchers can also now estimate single nucleotide polymorphism (SNP) heritability, the variance explained by the additive effects of common genetic variants tagged by a genotyping array (Yang et al. 2010, 2011). SNP heritability is expected to be less than twin and family-based heritability, since the former only estimates the additive effects of measured common variants, plus variants that are correlated (i.e. in linkage disequilibrium) with them, and ignores influences of DNA sequence differences that are rare and/or not well tagged by genotyping arrays. Since genome-wide association studies (GWAS) also only consider the additive effects of common variants, SNP heritability provides an estimate of the total genetic effect that could be identified with well-powered association studies of a given phenotype in a given population. Given the importance of SNP heritability, researchers have investigated approaches to maximise the accuracy of estimates, beyond increasing sample sizes or denser genotyping (van den Berg et al. 2014; Laurin et al. 2015; Cheesman et al. 2018; van der Sluis et al. 2010).

The dominant method for the estimation of SNP heritability, Genomic-RElatedness-based restricted Maximum-Likelihood (GREML), takes advantage of small genetic differences among many unrelated individuals to predict trait similarity (Yang et al. 2011). The effects of genetic variants that are not in linkage disequilibrium with common genotyped SNPs will not be captured using this method. However, when GREML is applied to family data, the higher genetic relatedness among relatives increases the correlation between genotyped SNPs and causal variants, because they are more likely to be inherited together (Zaitlen et al. 2013; Xia et al. 2016). This increase in linked variants helps to capture additional genetic variation not normally picked up in population studies of unrelated people, such as rare single nucleotide variants, copy number variants, and structural variants.

An extension of the method that uses family data, GREML-KIN, allows the estimation of two categories of genetic influence: population-level common variant heritability, plus additional heritability that is associated with kinship (Xia et al. 2016; Zaitlen et al. 2013). The first estimate is similar to that derived from GREML using unrelated individuals. The latter heritability estimate captures additional family-based effects, due to the increased correlation between genotyped SNPs and causal variants among relatives. The GREML-KIN method also allows for effects of environment sharing amongst family members, siblings and couples. This inclusion is important as it attempts to remove confounding that results from people who are more genetically related having more similar environments and higher phenotypic resemblance than people who are less related.

One study applied GREML-KIN to neuroticism and years of education (Hill et al. 2018) in Generation Scotland, a large family-based study (Smith et al. 2006). Neuroticism is a personality trait characterised by readily experiencing negative emotions. It is a strong predictor of common mental disorders, occupational attainment and mortality (Ormel et al. 2013). Years of education is also a complex trait that shows significant associations with diverse socioeconomic and health outcomes (Mackenbach et al. 2008). Twin studies have repeatedly demonstrated substantial broad sense heritability for neuroticism (40–60%) (Hettema et al. 2006) and educational attainment (40–50%) (Branigan et al. 2013). Extended twin family designs estimate the narrow sense heritability of neuroticism to be ~ 25% (Coventry and Keller 2005). These designs provide a better benchmark for additive genetic influence than the classical twin design, since they incorporate relatives of twin pairs to allow explicit separation additive genetic influences from non-additivity, shared environment, assortative mating, and gene-environment correlation. We are not aware of applications of the design to educational attainment, but one study estimated the narrow sense heritability of adult intelligence, a comparable phenotype, to be 44% (Vinkhuyzen et al. 2012). Neuroticism and educational attainment both index a range of important traits, and are available in numerous large datasets. As a result, they have been subject to some of the largest GWA studies of psychological traits (N = 449,484 for neuroticism (Nagel et al. 2018; Luciano et al. 2018); N = 1.1 million for years of education (Lee et al. 2018)). Nonetheless, only ~ 10% of the variance in neuroticism can be explained by the additive effects of common SNPs (Luciano et al. 2018; Nagel et al. 2018). Estimates of SNP heritability for years of education also fall substantially short of twin and pedigree estimates (14.7%; Lee et al. 2018).

GREML-KIN analyses in Generation Scotland revealed a large increase in heritability compared to a standard GREML analysis of unrelated individuals (Hill et al. 2018). For neuroticism, the total heritability from the best-fitting model was 30%, primarily accounted for by kin-based genetic effects (19%), as well as common variant effects tagged in studies of unrelated individuals (11%—akin to SNP heritability). They detected no family environment effects. For years of education, there was a strong kin genetic component (28%) in addition to common genetic influence (16%), plus substantial variance explained by sibling and couple similarity (11% and 31%, respectively). The findings align well with evidence from twin studies that the family environment influences education-related outcomes (~ 36% of the variance; Branigan et al. 2013) but not neuroticism (Polderman et al. 2015). If these results are replicated, then the total DNA-based heritability of neuroticism and education would be close to twin and pedigree study estimates. Moreover, a replication of these results would suggest that most of the variance in education (86%) can be captured with measured parameters. Notably, for each, the larger component of the genetic contributions results from less common variants not identified in genomic studies of unrelated individuals. The authors also found that rarer variants (0.1–1% in frequency) explained 12% of the variance in education, but did not influence variation in neuroticism (Hill et al. 2018).

Our study aimed to estimate familial influences on neuroticism and years of education in the UK Biobank, using GREML-KIN. We capitalise on the presence of thousands of family members in the UK Biobank to shed light on the genetic and environmental architecture of these two phenotypes. To robustly replicate the previous Generation Scotland study, we ensured that phenotype definitions were as similar as possible, and that there was no sample overlap. Based on previous research, we hypothesised that neuroticism and years of education would show increased heritability by exploiting the higher linkage disequilibrium within families. Our secondary analyses aimed to validate our kin-based estimates and specifically quantify the contribution of rarer genetic variants to the heritability of neuroticism and years of education. For this, we used the LDMS-I method, and stratified imputed genetic variants by their individual level of linkage disequilibrium and allele frequency to allow estimation of the variance explained by rarer variants (Evans et al. 2018).

## Methods

### Sample

Analyses were conducted using the UK Biobank, a resource containing rich phenotype and genotype data on ~ 500,000 individuals aged between 40 and 70 (Allen et al. 2014). To minimise confounding from population stratification, analyses were limited to white British individuals. We identified families and restricted heritability analyses to individuals with at least one family member in the UK Biobank, as well as phenotype data on neuroticism and/or years of education. Previous publications suggested a sample size of ~ 40,000 pairs of family members (parent-offspring, siblings, and couples) within the full dataset (Bycroft et al. 2018).

### Genotyping

Genome-wide genetic data from the full release of the UK Biobank data were collected and processed according to the quality control pipeline (Bycroft et al. 2018). For primary GREML-KIN analyses, we used genotyped or imputed SNPs with minor allele frequency > 0.01 and imputation confidence (INFO) score > 0.4, indicating well imputed variants. Due to computing memory constraints, we used PLINK2 to prune down to 241,678 variants in approximate linkage equilibrium using an r^2^ threshold of 0.2 (Chang et al. 2015) before calculating genetic relatedness matrices.

### Measures

Neuroticism was measured as a continuous trait, captured with 12 questionnaire items such as “Does your mood often go up and down?”, “Would you call yourself tense or ‘highly strung’?”. This trait was defined previously in the UK Biobank (Smith et al. 2013, 2016).

The years of education variable was defined according to ISCED categories, as in previous genomic studies in the UK Biobank and other samples (Hill et al. 2018; Lee et al. 2018). The six response categories were: none of the above (no qualifications) = 7 years of education; CSEs or equivalent = 10 years; O levels/GCSEs or equivalent = 10 years; A levels/AS levels or equivalent = 13 years; other professional qualification = 15 years; NVQ or HNC or equivalent = 19 years; college or university degree = 20 years of education. To test whether the number of response categories affected heritability estimates, as has been shown previously in the UK Biobank (Lee et al. 2018), we ran sensitivity analyses using a ‘coarsened’ years of education variable, plus a binary variable reflecting college completion (see Supplementary Fig. 1).

In all analyses the following covariates were included: age, sex, the first 40 ancestry principal components from the UK Biobank (Bycroft et al. 2018), genotyping batch, and assessment centre.

### Analyses

#### Identification of family members

Sibling and parent–offspring pairs were identified using relatedness files (KING n.d.) received with the UK Biobank data. Relatedness between two individuals is summarised by a kinship coefficient, which is defined as the probability that a random allele from an individual is identical by descent (IBD) with an allele at the same locus from the other individual (i.e. identical and inherited from a common ancestor). For example, in parent–offspring duos, kinship is ~ 0.25, as it is the probability that a random allele in a child is from one specific parent (0.5 since humans are diploid) multiplied by the probability that the parental allele from that parent is passed to the child (0.5; independent to the first probability). To allow for normal variation in within-pair similarity, first-degree relatives are therefore defined as pairs that have a pairwise kinship coefficient of ≥ 0.177 and ≤ 0.354.

**Fig. 1:**
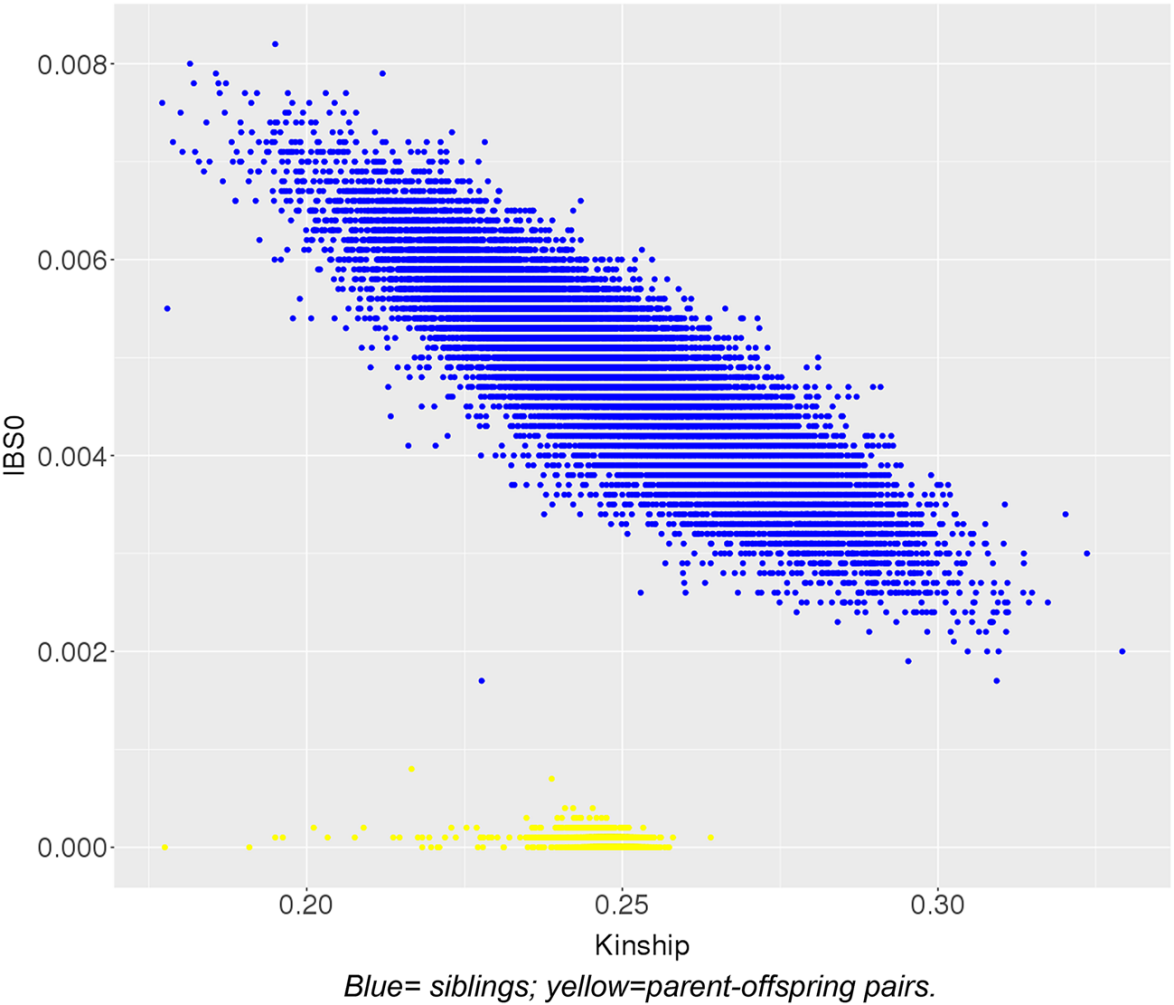
Kinship plotted against IBS0 for all first-degree relatives in the UK Biobank. Blue = siblings; yellow = parent–offspring pairs

To distinguish parent–offspring pairs from sibling pairs, we plotted the proportion of SNPs with *zero* identity-by-state (IBS0) within the kinship bounds of 0.177–0.354 (Fig. 1). IBS describes the probability that alleles are the same regardless of common ancestry. When comparing two individuals, variants are termed *IBS0* if neither allele is shared by the pair. Parent–offspring pairs have IBS0 = ~0 since they share one allele inherited by descent (IBD) in all positions on autosomes. In other words, an individual is unlikely to share *zero* variants with one of their parents, unless for example both copies come from the other parent (uniparental disomy), or unless there are genotyping errors meaning that shared variation is not called. In contrast, siblings have a higher pairwise IBS0.

Couples were identified as pairs of unrelated opposite-sex individuals matching exactly on a string of household variables: social deprivation (Townsend Deprivation Index), assessment centre, income, time at address, smoker in household, type of accommodation, relatives in household, number in household. This approach of matching on household variables was used in a recent study of assortative mating in the UK Biobank (Yengo et al. 2018). We note that there is potential for type 1 error: it is possible, especially in densely populated areas, that people could match on all eight variables by chance.

#### Kin-based SNP heritability method accounting for environmental similarity: GREML-KIN

GREML requires the calculation of genetic similarity for each pair of individuals across genotyped variants. This matrix of genomic similarity is compared to a matrix of pairwise phenotypic similarity using a random-effects mixed linear model, such that the variance of a trait can be decomposed into genetic and residual components, using maximum likelihood. Ordinarily, GREML is applied in samples of unrelated individuals and has a single common genetic variance component.

GREML-KIN is an extension of GREML that estimates the variance explained by multiple genetic and non-genetic sources. The method uses a linear mixed model to fit five matrices: G = common genotyped SNP effects; K = kin genetic effects; F = nuclear family (siblings, parent-offspring, and couples); S = siblings; C = couples. For the G matrix, we calculated genetic similarity for all possible pairs of individuals across all genotyped SNPs. As GREML-KIN allows for effects of the family environment, no relatedness cut-off was applied to the G matrix (unlike the standard GREML model applied only to unrelated individuals, where a cut-off of < 0.025 is typically used). The K matrix is a modified G matrix, containing only information on relatives (cut-off > 0.025), since values for unrelated pairs are set to 0. Family, sibling and couple (F, S and C) similarity matrices were created in the format required for GCTA. Elements in the genomic relatedness matrix were replaced by 0 if a pair did not have the specific relationship; and 1 if a pair do have the relationship, or for elements representing individuals’ relatedness to themselves.

Importantly, the variance components are not purely ‘genetic’ and ‘environmental’. The sibling and couple environment sharing matrices likely pick up variance due to other processes that inflate covariance between relatives, including dominance and assortative mating, respectively.

Assortative mating refers to greater similarity between partners than is expected by chance. This can result from multiple mechanisms, including direct choice based on phenotype, social homogamy, and convergence over time due to shared environments. Assortative mating amongst couples in the UK Biobank sample will be captured by fitting the couple similarity matrix (C). However, to the extent that phenotypic similarity among the parents of the UK Biobank participants reflects their genetic similarity, it is also likely that assortative mating in their parents will contribute to the additive genetic variance in our estimates (G + K). This is because assortative mating induces a positive correlation between trait-increasing alleles (‘gametic phase disequilbrium’), which elevates trait-specific genetic and phenotypic variance (Peyrot et al. 2016).

The genetic variance components are also likely to include some bias from the indirect effects of genetic variants shared with relatives. Genetic variants in the parents do not only have direct effects on offspring traits by being transmitted, but they also have indirect influences on offspring traits through the environment that they provide for their children. This can bias SNP-based heritability estimates (Young et al. 2018).

The residual component includes sources of variance that are not captured by the G, K, F, S or C matrices, particularly other environmental influences (idiosyncratic, individual-specific environments or perceptions that are not shared by family members) and error.

To identify the best-fitting model for each trait, we ran a model for every possible combination of variance components (31 models), and compared them with backwards stepwise likelihood ratio testing, starting with the full model and dropping non-significant parameters.

We compared GREML-KIN results against those from a standard GREML model in a subset of unrelated individuals from the family-based analyses. The standard GREML model uses a single genomic relatedness matrix with a cut-off to exclude one from each pair of related individuals (cut-off > 0.025). This approach therefore only detects population-level additive genetic effects tagged by common genotyped SNPs, plus potential confounding, for example from gene-environment correlation and population stratification. The residual component contains other sources of variance: gene–environment interaction, error, plus all of the environmental influence, rare variant effects that are not captured when using an unrelated population sample, and non-additive genetic effects.

#### GREML-LDMS-I to investigate the effects of less common variants

In our GREML-LDMS-I analyses, we started with whole genome data imputed from the HRC panel (93,095,623 autosomal variants; see Bycroft et al. 2018) We ran quality control to include variants across the allele frequency spectrum that were imputed with high confidence (INFO score > 0.80), and removed multiallelic variants. Three allele frequency bins were made, containing variants with minor allele frequency ranges of: 0.001–0.01, 0.01–0.1, 0.1–0.5, respectively. SNPs in each bin were split into high versus low linkage disequilibrium categories. We stratified by individual (rather than regional) SNP LD scores, since this has been shown to yield SNP heritability estimates that are more robust across different genetic architectures than estimates from other approaches (Evans et al. 2018). This led to six genome-wide genetic relatedness matrices, one for each allele frequency and LD bin (non-overlapping). All matrices included the same number of unrelated individuals (cut-off 0.025) with phenotype data and with at least one family connection as in standard GREML. The matrices were simultaneously fitted using a linear mixed model, and estimates were allowed to be negative. In supplementary analyses we explored whether the variance explained by the rarest alleles could be underestimated because their imputation quality was lower. Hence, we checked how many SNPs in each minor allele frequency bin were dropped when applying the INFO > 0.8 cutoff.

#### Sample independence

To ensure that our results were independent from the previous Generation Scotland study, we compared checksums for both samples to identify and remove overlapping participants. A checksum is the sum of nine numbers taken from binary genotype files. Checksums were obtained from Generation Scotland without accessing genotype data directly. We then ran checksums in the UK Biobank (after ensuring quality control of genomic data was the same), using a script from the Broad Institute, which is available online: https://personal.broadinstitute.org/sripke/share_links/checksums_download/outdated_readme/id_geno_checksum.v2.

We note that the number of relationships per individual in the UK Biobank is lower than in Generation Scotland. Their sample was selected to capture dense kinships, where many individuals have siblings, parents and spouses who are also study participants. This may result in lower power in the UK Biobank to detect influences of family similarity, especially if small in magnitude, and reduces power to separate confounding factors, as in biometric designs (McAdams et al. 2018).

#### Software

We used the following software in our analyses: identification of family members was performed using R; construction of genomic relationship matrices was done in GCTA; family, sibling and couple similarity matrices were made in bash; GREML analyses were conducted in GCTA. Scripts are available from the lead author on request. The UK Biobank is a public dataset available to all bona fide researchers (with funds to pay the access fee).

## Results

### Identification of family members

Columns 2–4 of Table 1 (bold) show how many pairs of the three types of family members we identified with available data on neuroticism and years of education. The numbers of family relationships closely matched findings from previous publications (Yengo et al. 2018; Bycroft et al. 2018). Column 5 contains the sample sizes of family pairs (the total of couple, sibling and parent–offspring pairs). From the number of family pairs, we derived the number of families (column 6), or in other words, the number of discrete sets of individuals who have at least one connection. The number of unique individuals (column 7) represents the total number of participants with at least one family member, after removing double-counted individuals who have multiple connections.

**Table 1:** Sample sizes for different family relationships in the UK Biobank

Phenotype	Couple (pairs)	Sib (pairs)	Parent-offspring (pairs)	Nuclear (pairs)	Families	Unique individuals
Neuroticism	16,451	14,562	4004	35,017	31,369	65,361
Education	23,201	21,564	5912	50,677	44,316	93,737

The discrepancies between the numbers of individuals, families and nuclear pairs reflects that most people only have one family member in the study. This contrasts to samples with dense kinship networks such as Generation Scotland, where many individuals have siblings, parents and spouses who are also study participants. As described in the Methods, we distinguished parent–offspring and sibling pairs according to their IBS0 (Fig. 1). We chose a threshold of IBS0 > 0.001 to define siblings (blue) separately from parent–offspring pairs (yellow).

Phenotypic correlations for neuroticism were 0.03 for couples, 0.14 for siblings, and 0.13 for parent–offspring pairs. Phenotypic correlations for years of education were 0.38 for couples, 0.30 for siblings, and 0.26 for parent–offspring pairs.

### Kin-based SNP heritability method accounting for environmental similarity: GREML-KIN

**Fig. 2:**
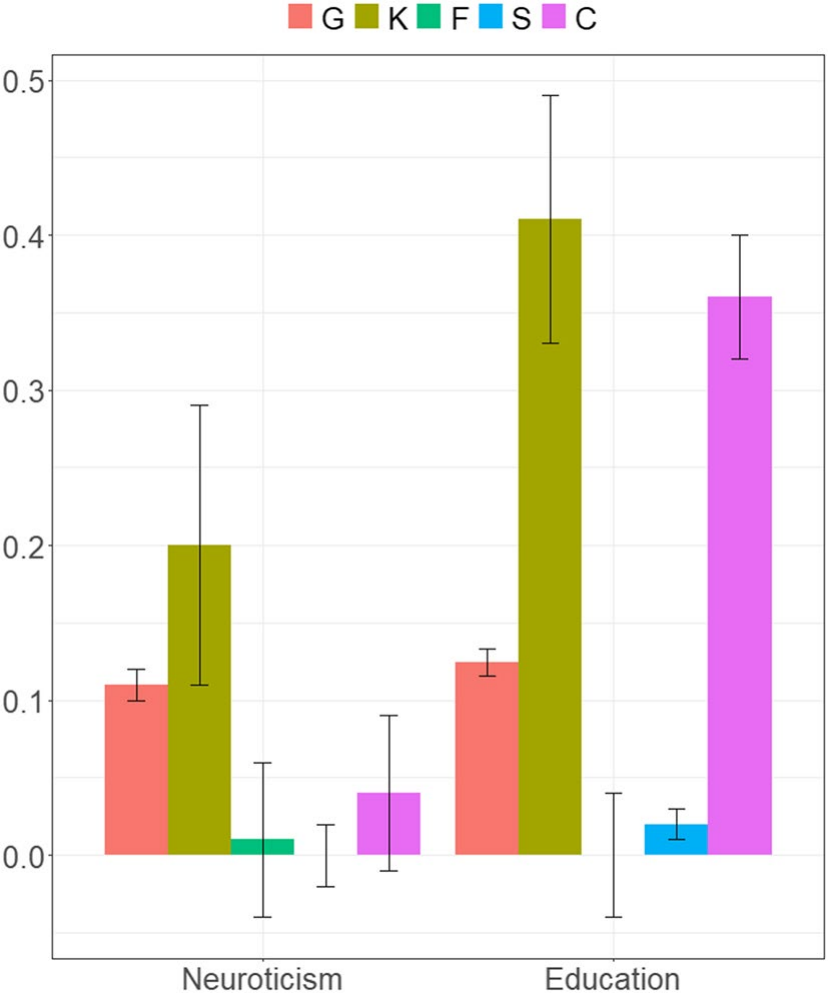
Variance component estimates for neuroticism and education, plus standard errors. *G* population-level effects of common genotyped SNPs; *K* kin-based genetic effects; *F* effects of nuclear family (siblings, parent-offspring, couple) similarity; *S* effects of sibling similarity; *C* effects of couple similarity. Note that for neuroticism, the estimates from our full model for the F, S, and C components are all non-significant, and standard errors cross zero. For education, the F and S components are non-significant

Figure 2 shows results for the full GREML-KIN models for neuroticism and education years. For neuroticism, the full model indicated that the variance explained by common genetic and kin-based variants is 11% (se = 0.01) and 20% (se = 0.09), respectively (31% in total, first two bars). Bars 3–5 demonstrate that there is no significant influence of family, sibling or couple similarity. For neuroticism, the selected model gives similar results and contains common SNP and kin-based genetic influences (11% (se = 0.01) and 16% (0.02) respectively). Compared to this best-fitting model, the inclusion of matrices to control for the influence of family environments (in the full model) does not reduce heritability. The total additive heritability of neuroticism when including relatives (27%, selected model) is substantially higher than in our analysis of unrelated individuals: 10% (se = 0.01; N = 44,694 unrelated individuals; Supplementary Table 1).

Figure 2 also shows the results for years of education from our full model. Due to computational memory limitations, it was necessary to run models for education in two parts (explained below). We report meta-analysed (inverse variance weighted means) results here. The results for each part are in Supplementary Tables 2 and 3. The heritability of years of education in our selected model was 56%, made up of 12% common SNPs at the population level (se = 0.01) and 44% kin-based effects (se = 0.02). This increases heritability considerably from 17% (se = 0.01) in unrelated individuals. The final model for education also contains a couple similarity effect of 35% (se = 0.01) in addition to the common and kin-based genetic influences.

See Supplementary Tables 1–3 for full model-fitting results, including all sub-models and fit statistics. As noted above, we ran two sets of GREML-KIN models in independent samples to reduce the computational burden. The first used the same matrices as in analyses of neuroticism. The second used new matrices including individuals who have education data and at least one family member, and who were not included in the neuroticism matrices. In defining these groups, we ensured that individuals in the same family were kept together.

Supplementary Fig. 1 gives GREML-KIN results for alternative education phenotypes (years of education with fewer categories, and degree/college completion). Estimates differed only slightly, and the conclusions remain the same.

### GREML-LDMS-I to investigate the effects of less common variants

Table 2 shows our estimates of the contribution of variants of different allele frequencies and individual linkage disequilibrium levels to neuroticism and years of education. For neuroticism, there is no evidence of a contribution of SNPs in the lowest minor allele frequency (MAF) bin (0.001–0.01), but SNPs of MAF 0.01-0.1 explained 3% of the phenotypic variance. All variants explained 11% (se = 0.02) of phenotypic variation. For education, variants tagged by low frequency SNPs (MAF between 0.001–0.01, and 0.01–0.1), particularly those in lower linkage disequilibrium, make a modest contribution to phenotypic variation, and all variants explained 21% of the phenotypic variance. Supplementary Table 4 shows that the lower the allele frequency bin, the more SNPs were dropped due to low imputation confidence.

**Table 2:** Results of GREML-LDMS-I variance components analyses for neuroticism and education using six minor allele frequency and LD bins

	MAF	0.001–0.01	0.001–0.01	> 0.01–0.1	> 0.01–0.1	> 0.1–0.5	> 0.1–0.5	
	LD	Lower	Higher	Lower	Higher	Lower	Higher	Total
	No. SNPs	1,772,407	1,772,399	1,756,294	1,756,290	2,105,014	2,104,972	11,267,376
Neuroticism	h2	0.00 (0.02)	0.00 (0.01)	0.02 (0.01)	0.01 (0.03)	0.05 (0.01)	0.04 (0.004)	0.11 (0.02)
Education	h2	0.06 (0.02)	0.00 (0.05)	0.03 (0.01)	0.01 (0.01)	0.07 (0.01)	0.05 (0.03)	0.21 (0.02)

*MAF* minor allele frequency, *LD* linkage disequilibrium, *h2 (se)* variance explained by SNPs in MAF and LD bin, plus standard error

Checksum analyses indicated that there were no family members with phenotype data in the UK Biobank who were also in Generation Scotland. We can therefore be confident that our results are independent of the previous study (Hill et al. 2018).

## Discussion

In this study, we capitalised on the family information and genome-wide genotypes in the UK Biobank to estimate genetic and family environmental influences on neuroticism and years of education. Inclusion of family-based genetic effects substantially increases the heritabilities of neuroticism and education from 10 and 17% in standard analyses of unrelated individuals, to 27% and 56% respectively. Our estimates closely replicate previous findings from an independent sample (Generation Scotland; Hill et al. 2018). The additional family-based influences likely include rare variants, copy number variants, and structural variants. Turning to our non-genetic findings, we did not detect any influence of nuclear family, sibling or couple similarity on variation in neuroticism. This is consistent with evidence against shared environmental contributions to personality (Nivard et al. 2015; Hettema et al. 2006; Coventry and Keller 2005), and with the previous study using the same method (Hill et al. 2018). For education years, we found that 35% of the variance was explained by couple similarity. This couple effect likely captures assortative mating (education years is likely to have occurred before they became couples) and family environment effects on education.

At least three potential biases mean that the higher GREML-KIN heritability may not only be explained by ‘revealing additional genetic effects’. First, confounding is likely to remain in the heritability estimates due to indirect effects of alleles shared by parents and offspring (passive gene-environment correlation). Simulations in an Icelandic dataset suggest that although GREML-KIN gives unbiased heritability estimates when family environmental influences are present, it over-estimates heritability if phenotypes are also substantially influenced by passive gene-environment correlation (Young et al. 2018). The influence of passive gene-environment correlation for educational attainment in the UK Biobank is suggested by the lower SNP heritability for adoptees, whose rearing environments are less correlated with their genotypes (Cheesman et al., 2019). In the future, methods that can distinguish direct from indirect influences should be applied to neuroticism, education and other complex, socially-contingent traits (Eaves et al. 2014; Visscher et al. 2006; Young et al. 2018).

Second, geographic population differences complicate our interpretations of heritability estimates, particularly for education. One study found that controlling for urban childhood residence, a proxy for a range of environmental factors, reduced the SNP heritability of education (but not the heritabilities of height or body mass index) above and beyond other controls for population stratification (Conley et al. 2014). Stratification in the UK Biobank is present even at a fine scale, is unlikely to be completely removed by controlling for principal components, and is potentially more severe in rare variant analyses (Haworth et al. 2019; Young 2019).

A third key issue is assortative mating. There is clear evidence for assortment on educational attainment, including in the UK Biobank (Hugh-Jones et al. 2016; Robinson et al. 2017; Yengo et al. 2018). Our estimates of additive genetic variance are likely to be overestimated due to assortative mating among the parents of the UK Biobank participants (see Methods). Even beyond this, assortative mating may inflate the estimated additive genetic variance because it increases phenotypic covariance among relatives and the covariance among specific trait-associated loci, but it is not captured by genome-wide genetic relatedness (the predictor in our GREML models). Assortative mating may also exacerbate the bias due to gene-environment correlation (Keller et al. 2009; Kong et al. 2018). Specifically, the effects of variants influencing educational attainment in offspring may be magnified because variants are also present in the parents, influencing parental education, parents’ choice of a mate with similar attainment, and the rearing environment they provide. The effects of assortment on variance component estimates from genomic heritability methods are likely to be complicated and are an important area for future research.

Although the potential biases mean that interpretations should be cautious, our heritability estimates are notably closer to twin and extended twin family study estimates than are those from standard GREML analysis. The latter design is able to differentiate the effects of additive genetics common environment, assortative mating and gene-environment correlation, and the best-powered heritability estimate for neuroticism of ~ 25% (Coventry and Keller 2005) is close to ours (27%). On the other hand, our total heritability estimate for educational attainment (56%) is notably high and likely to be more strongly biased. It is at the upper end of twin estimates in the literature (40–50%), even though twin heritability does not only reflect additive genetic effects. The increase beyond twin heritability is likely to be partly due to passive gene-environment correlation, which inflates the shared environment component rather than heritability in twin studies.

Our allele frequency and LD partitioned heritability results (GREML-LDMS-I) give mixed support for the notion that the contribution of family-associated genetic influence is explained by rarer variants. For education, some effects of low allele frequency, low LD variants are captured by our partitioned genomic relatedness matrices. For neuroticism, the same GREML-LDMS-I heritability analyses indicated less rare genetic influence than expected based on the large kin-genetic component from the GREML-KIN model-fitting. For neuroticism, the gain in heritability from GREML-KIN despite the lack of variance in the 0.001-0.01 frequency bins is perplexing but may be partly explained by poor imputation,very rare and structural variation, and population stratification. With regard to imputation quality, the rarest SNPs were more likely to be dropped prior to analysis (see Supplementary Table 4). Since rare SNPs tag other rare SNPs poorly, dropping rare ones would lead to more dramatic underestimation of their effects.

Evidence from other studies suggests an important role for rarer variants, which will be further elucidated with the increasing availability of whole genome sequence data. Multifactorial traits such as personality and educational attainment are thought to have complex genetic aetiologies, consisting of interplay between common and rare variation. Recent exome sequencing work has demonstrated rare coding variant effects on many psychiatric traits [that are closely related to neuroticism; (Ganna et al. 2018)]. Other evidence for interactions between common variants across the genome and rare copy number variants in schizophrenia (Bergen et al. 2018) shows the importance of examining different types of genetic variation together. Importantly, pedigree estimates of the heritability of height and BMI can be recovered by using whole genome sequence data (rather than imputed data) in GREML analyses (Wainschtein et al. 2019). However, much larger sample sizes are needed to detect rare genetic influences, and confounding factors such as stratification, indirect effects and assortative mating are still present.

The additional influences correlated with kinship detected in this study indicate a chance to improve prediction using polygenic scores. Prediction accuracy is a function of the SNP heritability of the target sample, and of the SNP heritability of, and genetic correlation with, the GWAS discovery phenotype (de Vlaming et al. 2017). Polygenic scores for neuroticism currently explain maximum 4.2% of the phenotypic variance in independent samples, which is a fraction of the common SNP heritability (~ 10%; Nagel et al. 2018). For years of education, polygenic scores explain > 10% of the phenotypic variance, close to the SNP heritability of 15%. Increasing sample sizes and phenotypic homogeneity for GWAS of unrelated population samples will continue to narrow this gap between polygenic prediction and SNP heritability. However, the population-level common variant approach may be limited for educational attainment, given the relatively high variance already explained by polygenic scores. For both traits, genomic prediction could be improved by leveraging family information (Lee et al. 2017). Inclusion of relatives could help to capture additional effects of typically untagged variants that are rare and possibly even family-specific. Importantly, for the purpose of prediction, genetic scores need not be, and already are not, ‘pure’ indices of individual genetic propensity. It may help to tag influential aspects of the environment, for example by combining parent and child polygenic scores within a single model.

In summary, we provide evidence for substantial family-based and common genetic effects on neuroticism and years of education in the UK Biobank. These results motivate the recruitment of samples with dense kinships and methods to leverage genomic family information, whilst understanding gene-environment interplay.

## Electronic supplementary material

Below is the link to the electronic supplementary material.

Supplementary material 1 (DOCX 50 kb)
